# Pretreatment with Total Flavonoid Extract from *Dracocephalum Moldavica* L. Attenuates Ischemia Reperfusion-induced Apoptosis

**DOI:** 10.1038/s41598-018-35726-4

**Published:** 2018-11-30

**Authors:** Cheng Zeng, Wen Jiang, Xiaoyi Yang, Chenghui He, Wen Wang, Jianguo Xing

**Affiliations:** 1 College of Pharmacy, Xinjiang Medical University, No. 393 Xinyi Road, Urumqi, 830054 China; 2 Xinjiang Institute of Materia Medica, No. 140 Xinhua South Road, Urumqi, China; 3 Department of Experimental Animal Center, Xuanwu Hospital of Capital Medical University, No. 45 Changchun Street, Beijing, China

## Abstract

We previously demonstrated the cardio-protection mediated by the total flavonoid extracted from *Dracocephalum moldavica* L. (TFDM) following myocardial ischemia reperfusion injury (MIRI). The present study assessed the presence and mechanism of TFDM-related cardio-protection on MIRI-induced apoptosis *in vivo*. Male Sprague-Dawley rats experienced 45-min ischemia with 12 h of reperfusion. Rats pretreated with TFDM (3, 10 or 30 mg/kg/day) were compared with Sham (no MIRI and no TFDM), MIRI (no TFDM), and Positive (trapidil tablets, 13.5 mg/kg/day) groups. In MIRI-treated rats, high dose-TFDM (H-TFDM) pre-treatment with apparently reduced release of LDH, CK-MB and MDA, enhanced the concentration of SOD in plasma, and greatly reduced the infarct size, apoptotic index and mitochondrial injury. H-TFDM pretreatment markedly promoted the phosphorylation of PI3K, Akt, GSK-3*β* and ERK1/2 in comparison with the MIRI model group. Western blot analysis after reperfusion also showed that H-TFDM decreased release of Bax, cleaved caspase-3, caspase-7 and caspase-9, and increased expression of Bcl-2 as evident by the higher Bcl-2/Bax ratio. TFDM cardio-protection was influenced by LY294002 (PI3K inhibitor) and PD98059 (ERK1/2 inhibitor). Taken together, these results provide convincing evidence of the benefit of TFDM pretreatment due to inhibited myocardial apoptosis as mediated by the PI3K/Akt/GSK-3*β* and ERK1/2 signaling pathways.

## Introduction

Ischemia heart disease (IHD) is one of most lethal coronary heart diseases and remains a grave public health threat^1,2^. According to the World Health Organization, IHD accounted for approximately 7.6 million deaths in 2012. IHD is predicted to be the second major cause of global rural and urban death by 2030^3^.

The only available therapy for IHD is prompt-coronary reperfusion to restore blood flow to the ischemic myocardium. This reduces cell death and alleviates myocardial dysfunction^4,5^. Paradoxically, rapid reperfusion can be deleterious, as it induces the death of cardiomyocytes^6,7^. Mechanistically, reperfusion increases the production of oxygen free radicals and enhances myocardial apoptosis^8,9^.

Cardiomyocyte death is a common characteristic of MIRI. Apoptosis is the principal cellular pathway which results in cardiomyocyte death^10^. In MIRI, significant myocardial cell damage can be resulted from excessive apoptosis^11^. Heart damage induced by MIRI can be minimized by the prevention of apoptosis. The activation of a class of aspartate-specific cysteine proteases known as caspases is a key feature of apoptosis^12,13^. Apoptosis is principally mediated by two pathways^14^. One involves the mitochondria. Mitochondria-induced apoptosis is significant role in the pathogenesis of MIRI^15^. The other pathway is the phosphatidylinositol-3-kinase (PI3K)/Akt pathway. Activation of this pathway reduces myocardial apoptosis, which is important in maintaining mitochondrial integrity by the phosphorylation of proteins including glycogen synthase kinase 3*β* (GSK-3*β*)^16,17^. A variety of drugs target GSK-3*β* in bestowing myocardial protection^18,19^. The extracellular signal-regulated kinase 1/2 (ERK1/2) signaling pathway is activated in response to various cytokines and growth factors and mediate primarily mitogenic and anti-apoptotic signals. Akt can phosphorylate GSK-3*β* and ERK1/2, and the phosphorylated molecules are cardioprotective against MIRI^20,21^.

Pharmacologically mitigating MIRI may be a viable means to treat MIRI. Some flavonoids are the most effective biologically active compounds in plants. These compounds might be useful as chemopreventive and therapeutic agents for cardiovascular disease^22^. The cardioprotective effect of flavonoids is attributed to their anti-oxidant, anti-inflammatory and anti-apoptotic properties^23^. *Dracocephalum moldavica* L. (also known as Xinjiang in China) is an annual herbaceous plant that is enriched in flavonoids^24–26^. In addition, recently, it has been demonstrated to be effective in treating cardiovascular disease, fatigue insomnia, upset, hypertension and heart failure^27,28^.

**Figure 1:**
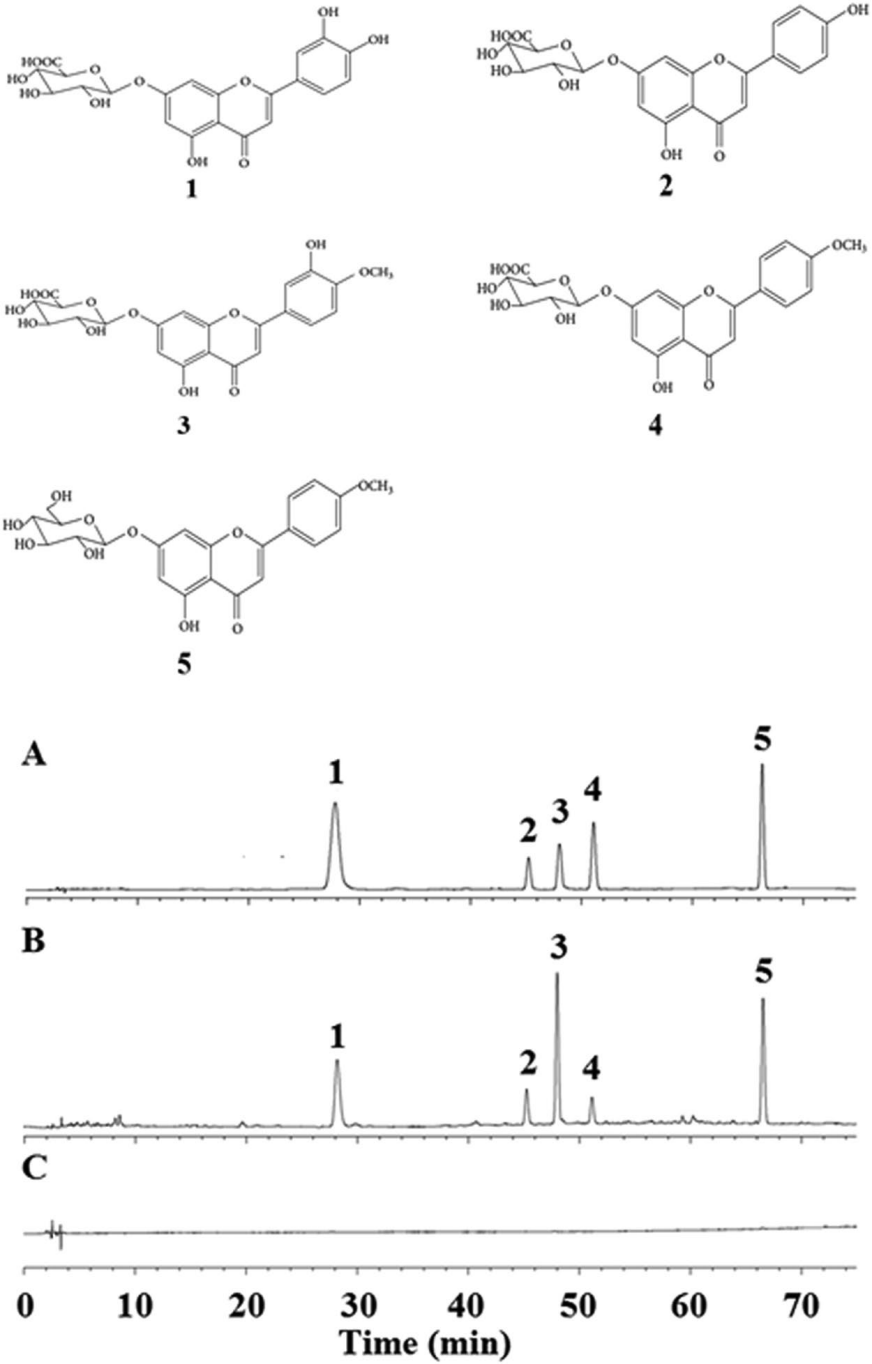
Chemical structures and HPLC chromatograms of five components of TFDM. (**A**) Standard mix controls; (**B**) TFDM sample; (**C**) Blank. 1. Luteolin-7-O-β-D-glucuronide; 2. Apigenin-7-O-β-D-glucuronide; 3. Diosmetin-7-O-β-D-glucuronide; 4. Acacetin-7-O-β-D-glucuronide; 5. Tilianin).

We have previously demonstrated the utility of total flavonoid extract from *Dracocephalum* *moldavica* L. (TFDM) to protect cardiomyocytes and have shown its’ anti-oxidant activity^29,30^. Very recently, we reported the five main chemical constituents of TFDM^24^, which include luteolin-7-*O*-β-D-glucuronide^31^, apigenin-7-*O*-β-D-glucuronide^32^, diosmetin-7-O-β-D-glucuronide^33^, acacetin-7-O-β-D-glucuronide^34^ and tilianin^35^. These compounds (Fig. 1) all display significant myocardial protective effects. While TFDM can activate the PI3K/Akt signaling pathway to reduce apoptosis, whether TFDM inhibits apoptosis during MIRI via GSK-3*β*-dependent cell-survival and the ERK1/2 signaling pathway is unknown.

The current work has three aims. The first is to investigate whether TFDM exerts any myocardial protection effect against MIRI. The second is to measure whether TFDM reduces oxidation and myocardial injury in MIRI rats. The third is to elucidate the mechanisms of TFDM on cardiomyocyte apoptotic pathways (PI3K/Akt/GSK-3*β* and ERK1/2 signaling pathway) in the MIRI. The data will provide clinical references and the basis for developing novel potential natural drugs active when treating MIRI.

## Results

### Myocardial biochemical markers determination

In comparison with the Sham group, LDH, MDA and CK-MB levels in the MIRI group was increased, whereas SOD activity was decreased (*P* < 0.01). When compared with the MIRI group, the activities of LDH and CK-MB, and MDA level were significantly decreased in high dose-TFDM (H-TFDM) and trapidil tablets groups. However, SOD activity was elevated (*P* < 0.01) (Table 1).

**Table 1:** Influence of TFDM on LDH, CK-MB, SOD and MDA in rat hearts after myocardial ischemia reperfusion (n = 10).

Group	LDH (U/mL)	CK-MB (ng/mL)	SOD (U/mL)	MDA (nmol/mL)
Sham	48.03 ± 5.18	2.28 ± 0.37	23.27 ± 3.49	3.18 ± 0.33
MIRI	289.96 ± 41.51^△△^	6.22 ± 0.81^△△^	12.53 ± 2.35^△△^	8.82 ± 1.57^△△^
L-TFDM	255.58 ± 25.44^*^	5.92 ± 0.42	13.57 ± 1.84^*^	7.94 ± 0.25
M-TFDM	128.38 ± 6.52^**^	3.54 ± 0.19^*^	18.85 ± 2.55^**^	4.54 ± 0.37^*^
H-TFDM	61.15 ± 5.84^**^	2.75 ± 0.25^**^	22.58 ± 3.11^**^	3.11 ± 0.18^**^
Control drug	56.68 ± 8.76^**^	2.40 ± 0.13^**^	23.61 ± 2.18^**^	2.98 ± 0.29^**^

^△△^*P* < 0.01 vs Sham: ^*^P < 0.05, ^**^P < 0.01 vs MIRI.

### Infarct Size and Histological & Examination (H&E)

**Figure 2:**
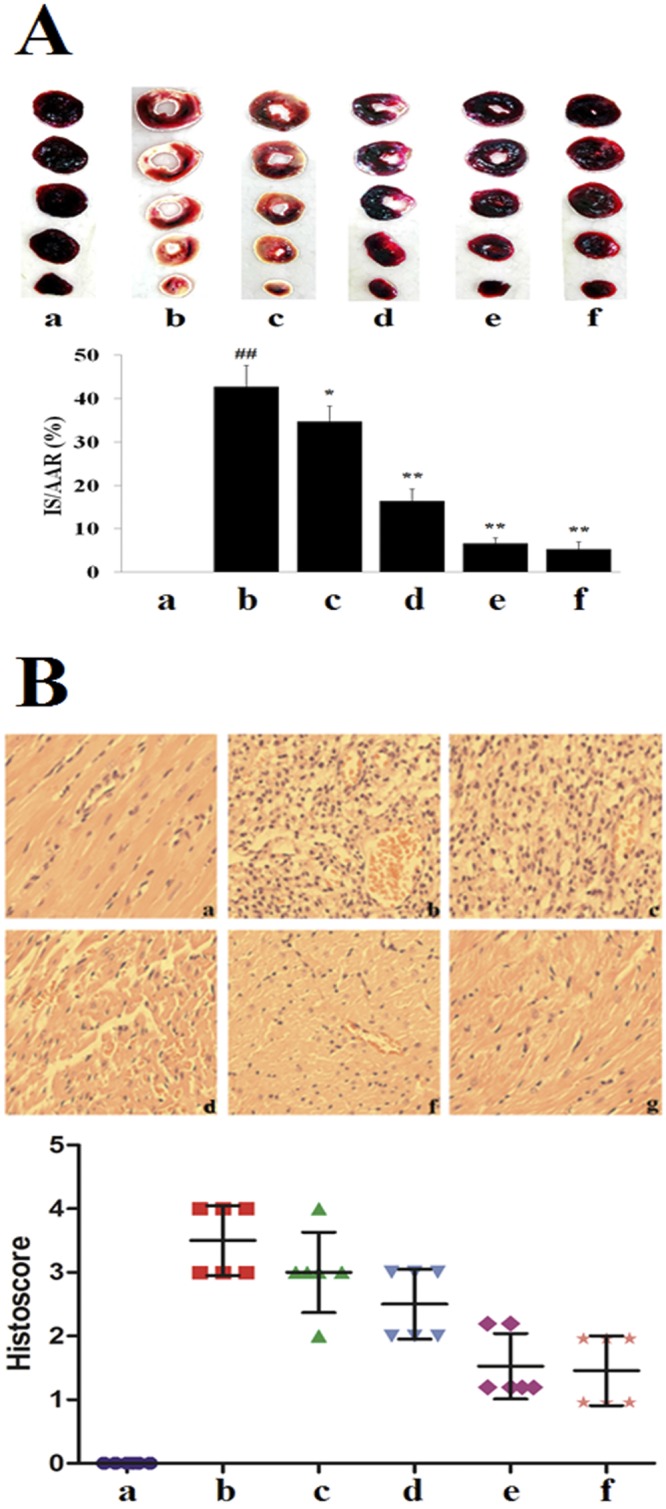
Myocardial infarct size and histological characterization in MIRI rats. (**A**) Images of TTC stained heart sections, and the quantitative analysis, TTC stains living tissue as deep red color while necrotic tissue is TTC negative and appears as white color. The infarcted area is marked with a red line (a: Sham; b: MIRI; c: L-TFDM; d: M-TFDM; e: H-TFDM; f: Control drug, ^##^*P* < 0.01; ^*^P < 0.05; ^**^P < 0.01, n = 10). (**B**) Histological characterization in the hearts of MIRI rats. Magnification ×40 (A, a: Sham; b: MIRI; c: L-TFDM; d: M-TFDM; e: H-TFDM; f: Control drug) (Scatter plot of histoscore. n = 10 rats in each group).

In Fig. 2A, there was scarce percent of infarct in the sham group. Compared with the sham group, there was larger myocardial infarction size in the MIRI group (*P* < 0.01). On the contrary, compared with the MIRI group, the myocardial infarction sizes were decreased by pretreating with various concentrations of TFDM. Moreover, the result of H-TFDM was similar to that of positive drug (*P* > 0.05).

The Sham group displayed normal structure of the myocardium, whereas the MIRI group revealed marked membrane damage of cardiomyocytes with extensive edema, necrosis, and inflammatory cell infiltration (Fig. 2B). Pre-administration of H-TFDM and positive drug could avoid the damage of cardiac cell membrane which was occasionally accompanied by myofiber loss and inflammatory cell infiltration. Cellular integrity was nearly identical with cells of the Sham group. In contrast to the Sham group, analysis of MIRI group revealed an obvious enhanced MIRI-induced injury.

### Effect of TFDM on Myocardium Apoptosis

**Figure 3:**
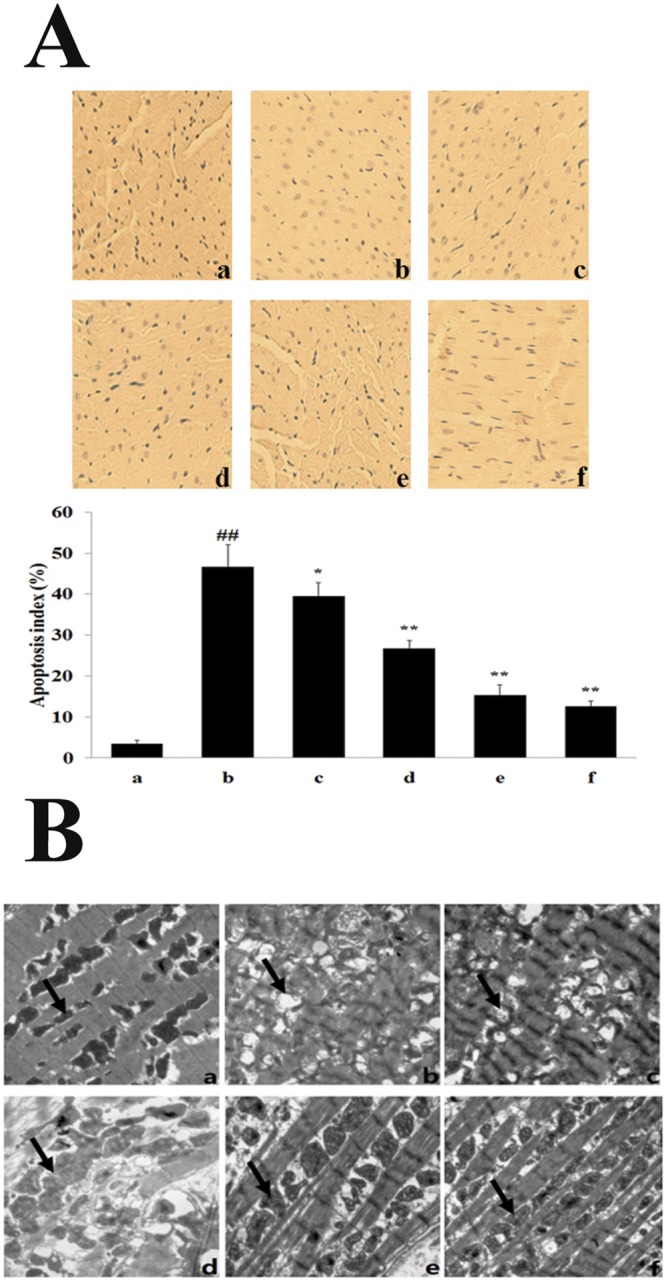
Cardiomyocytes apoptosis and morphological changes of myocardial tissue. (**A**) Analysis of apoptosis in the hearts. TUNEL positive cell ratio (a–f, represent results of Sham, MIRI, L-TFDM, M-TFDM, H-TFDM and Control drug group, respectively, ^##^*P* < 0.01; ^*^P < 0.05; ^**^P < 0.01, n = 10). (**B**) The morphological changes of myocardial tissue by transmission electron microscope (×15000) in the Sham group (a), MIRI group (b), L-TFDM (c), M-TFDM (d), H-TFDM (e) and control drug (f), black arrows indicate individual mitochondrion. In Sham group, normal myocardial ultrastructure is shown in a. After 12 h of reperfusion, grossly distorted structures of myofibrils and mitochondria are noted in b, while better-preserved myofibrillar and mitochondrial ultrastructure are shown in hearts of H-TFDM group (e) and positive drug group (f).

No obvious apoptosis was evident in the Sham group, whereas the number of apoptotic cells was increased in the MIRI group (Fig. 3A). In contrast, few apoptotic cell nuclei were confirmed in the H-TFDM or control drug pretreated groups. Pretreatment with H-TFDM and positive drug revealed significantly decreased numbers of positive nuclei in comparison with the MIRI group (*P* < 0.01). There existed no obvious difference in the numbers of apoptotic cells in H-TFDM and control drug pretreated rats (P > 0.05).

### Myocardial Mitochondrial Ultrastructure

As shown in Fig. 3B, the ultrastructure of the cardiac muscle from rats of the sham, H-TFDM and positive groups was identical, the myofilaments typically exhibited an ordered arrangement; the mitochondria was intact with a round or ovoid shape and an neatly arrangement, and the decidua were tightly connected. However, myocardial mitochondria from the model group had suffered substantial structural damage, with the myofilaments dissolved or even broken; the mitochondria were significantly swollen and crista space was widened and broken. In the low dose-TFDM (L-TFDM) and middle dose-TFDM (M-TFDM) groups, the myofilaments exhibited an ordered arrangement; some myofilaments and sarcomere spaces were widened and dissolved, but the majority of the mitochondria had an intact morphology with clear and visible cristae. In addition, some mitochondria were slightly swollen.

### Western Blot Analysis

**Figure 4:**
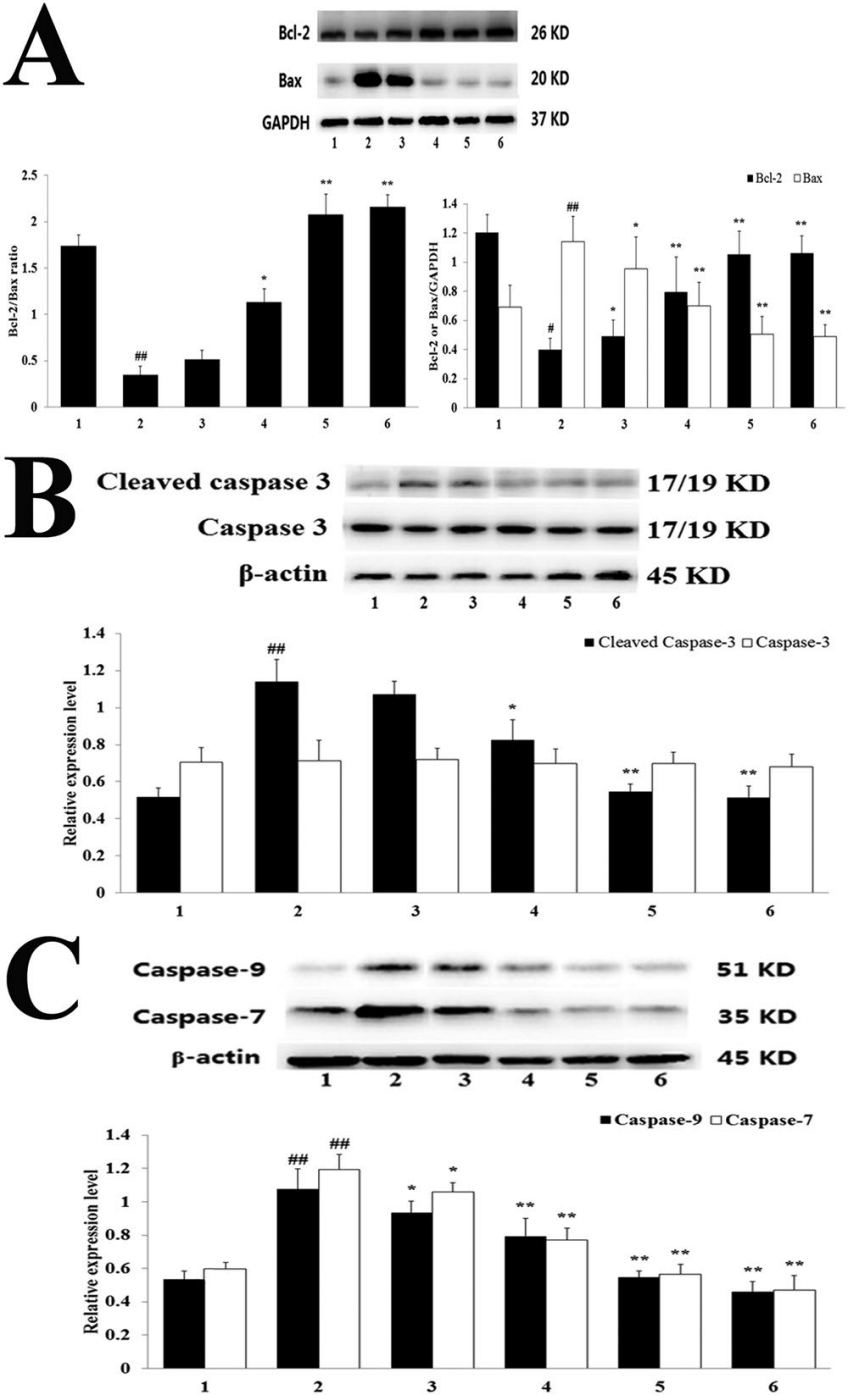
Bcl-2 and Bax expression, Cleaved-caspase-3 and caspase-3 expression and Caspase-7 and Caspase-9 expression in MIRI rats. (**A**) Bcl-2 and Bax assay (1–6, represent results of Sham, MIRI, L-TFDM, M-TFDM, H-TFDM, control drug group, respectively, ^##^*P* < 0.01; ^*^P < 0.05; ^**^P < 0.01, n = 10). (**B**) Cleaved-caspase-3 and caspase-3 assay (1–6, represent results of Sham, MIRI, L-TFDM, M-TFDM, H-TFDM, control drug group, respectively, ^##^*P* < 0.01; ^*^P < 0.05; ^**^P < 0.01, n = 10). (**C**) Caspase-7 and Caspase-9 assay (1–6, represent results of Sham, MIRI, L-TFDM, M-TFDM, H-TFDM, control drug group, respectively, ^##^*P* < 0.01; ^*^P < 0.05; ^**^P < 0.01, n = 10).

Bcl-2 protein expression and the proportion of Bcl-2 and Bax were significantly elevated in the H-TFDM and control drug groups, in contrast to the MIRI group, whereas cleaved caspase-3 and Bax expression was decreased (*P* < 0.01). Caspase-3 displayed no definite difference in each group (*P* > 0.05; Fig. 4A,B). An obvious reduction in the expression of caspase-7 and caspase-9 protein expression was detected in the H-TFDM and trapidil tablets groups, in contrast to the MIRI group (*P* < 0.01; Fig. 4C).

**Figure 5:**
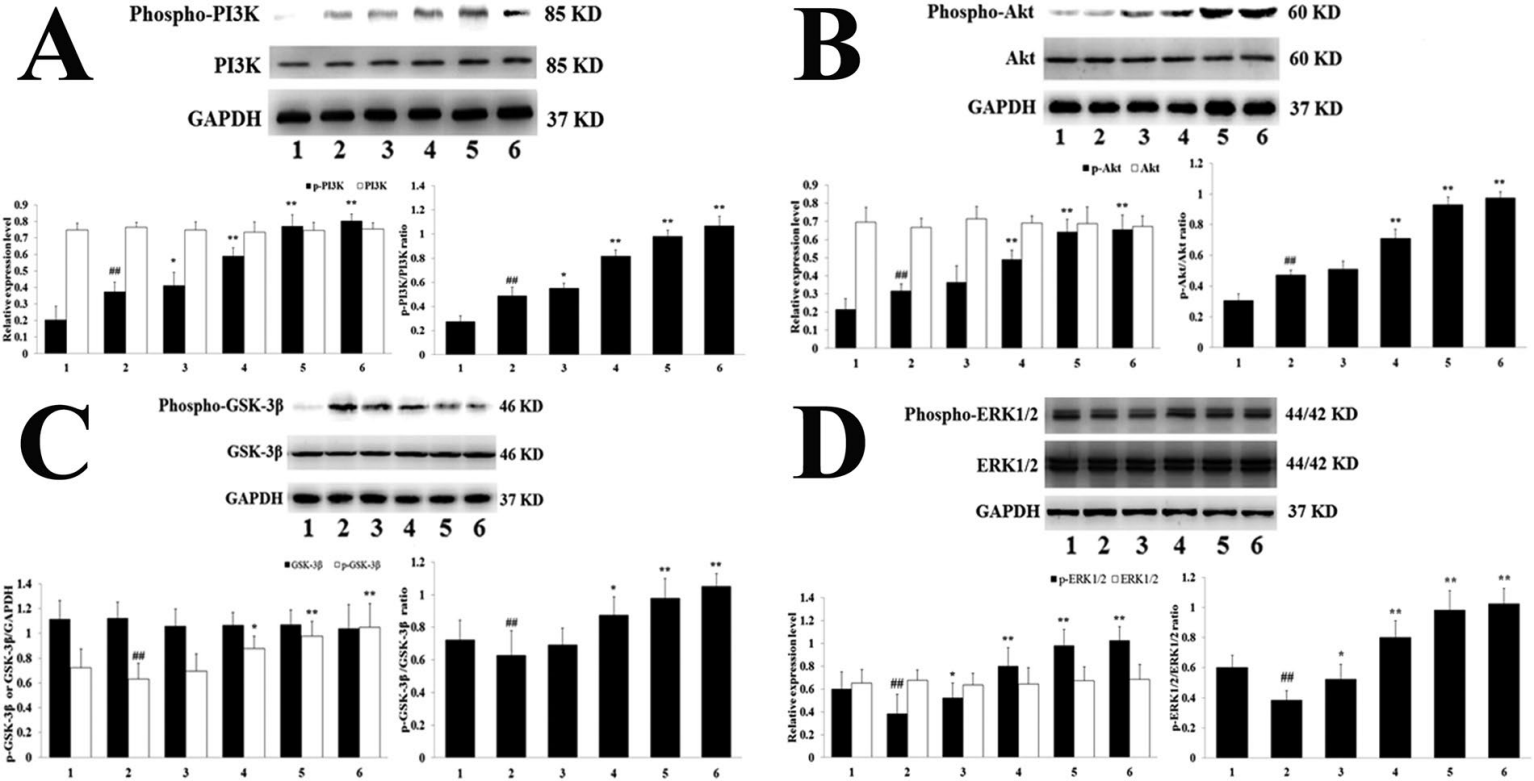
Phospho-PI3K and PI3K expression, Phospho-Akt and Akt expression, Phospho-GSK-3*β* and GSK-3*β* expression and Phospho-ERK1/2 and ERK1/2 expression in MIRI rats. (**A**) Phospho-PI3K and PI3K assay (1–6, represent results of Sham, MIRI, L-TFDM, M-TFDM, H-TFDM, control drug group, respectively, ^##^*P* < 0.01; ^*^P < 0.05; ^**^P < 0.01, n = 10). (**B**) Phospho-Akt and Akt assay (1–6, represent results of Sham, MIRI, L-TFDM, M-TFDM, H-TFDM, control drug group, respectively, ^##^*P* < 0.01; ^*^P < 0.05; ^**^P < 0.01, n = 10). (**C**) Phospho-GSK-3*β* and GSK-3*β* assay (1–6, represent results of Sham, MIRI, L-TFDM, M-TFDM, H-TFDM, control drug group, respectively, ^##^*P* < 0.01; ^*^P < 0.05; ^**^P < 0.01, n = 10). (**D**) Phospho-ERK1/2, and ERK1/2 assay (1–6, represent results of Sham, MIRI, L-TFDM, M-TFDM, H-TFDM, control drug group, respectively, ^##^*P* < 0.01; ^*^P < 0.05; ^**^P < 0.01, n = 10).

The protein expression levels of PI3K phosphorylation and AKT phosphorylation, and the PI3K phosphorylation/PI3K and Akt phosphorylation/Akt ratios were elevated in the H-TFDM and control drug groups, in contrast to the MIRI group (Fig. 5A,B), whereas no apparent effect was evident in the level of total Akt and PI3K (*P* < 0.01). The H-TFDM and control drug groups displayed obvious elevations in the expression of GSK-3*β* phosphorylation and ERK1/2 phosphorylation, in contrast to MIRI group (*P* < 0.01; Fig. 5C,D).

**Figure 6:**
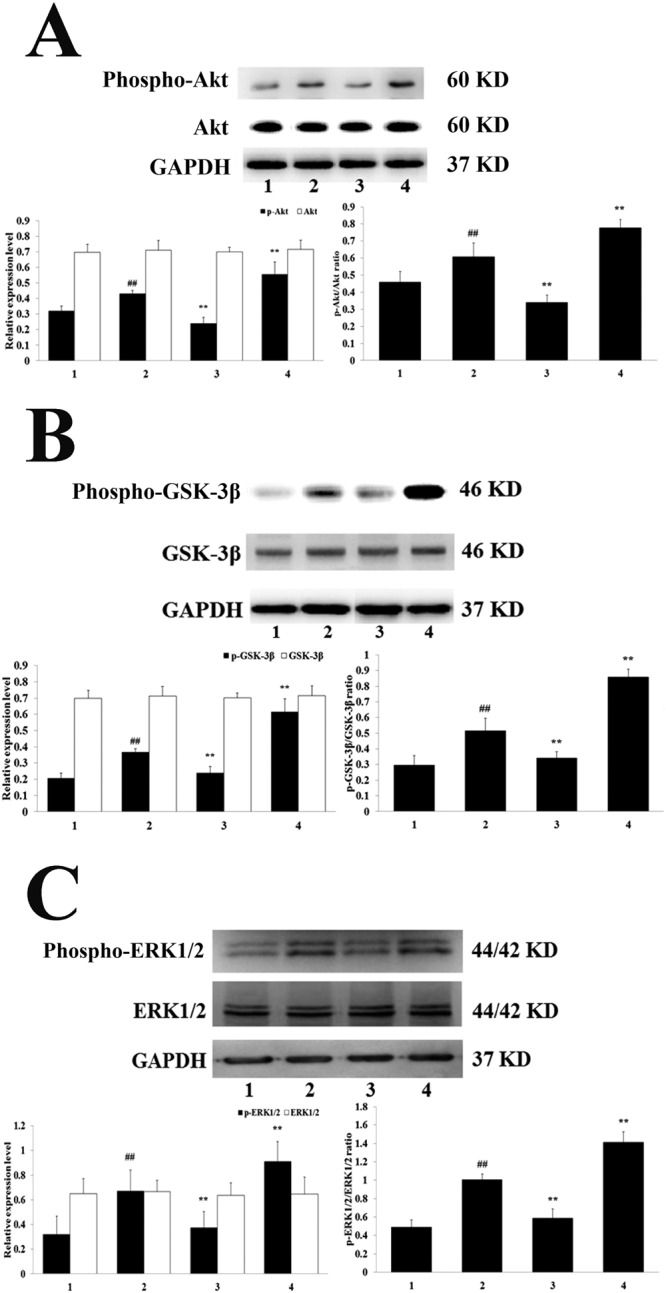
Phospho-Akt and Akt expression, Phospho-GSK-3*β* and GSK-3*β* expression and Phospho-ERK1/2 and ERK1/2 expression in MIRI rats. (**A**) Phospho-Akt and Akt assay (1–4, represent results of Sham, MIRI, H-TFDM + MIRI + LY294002, H-TFDM + MIRI group, respectively, ^##^*P* < 0.01; ^*^P < 0.05; ^**^P < 0.01, n = 10). (**B**) Phospho-GSK-3*β* and GSK-3*β* assay (1–4, represent results of Sham, MIRI, H-TFDM + MIRI + LY294002, H-TFDM + MIRI group, respectively, ^##^*P* < 0.01; ^*^P < 0.05; ^**^P < 0.01, n = 10). (**C**) Phospho-ERK1/2 and ERK1/2 assay (1–4, represent results of Sham, MIRI, H-TFDM + MIRI + LY294002, H-TFDM + MIRI group, respectively, ^##^*P* < 0.01; ^*^P < 0.05; ^**^P < 0.01, n = 10).

In addition, an obvious elevation in these proteins occurred in the H-TFDM group (*P* < 0.01). The up-regulation in the H-TFDM group was blocked by LY294002 administration (*P* < 0.01). However, there were no obvious differences between the MIRI + H-TFDM + LY294002 and MIRI groups concerning Akt phosphorylation and GSK-3*β* phosphorylation (*P* > 0.05 vs MIRI) (Fig. 6A,B). Similarly, the cardio-protection of TFDM was blocked by treatment with PD98059, an inhibitor of ERK1/2, in contrast to the MIRI + TFDM group *P* < 0.01; Fig. 6C).

### Long-term Effects of TFDM on Myocardial Fibrosis

**Figure 7:**
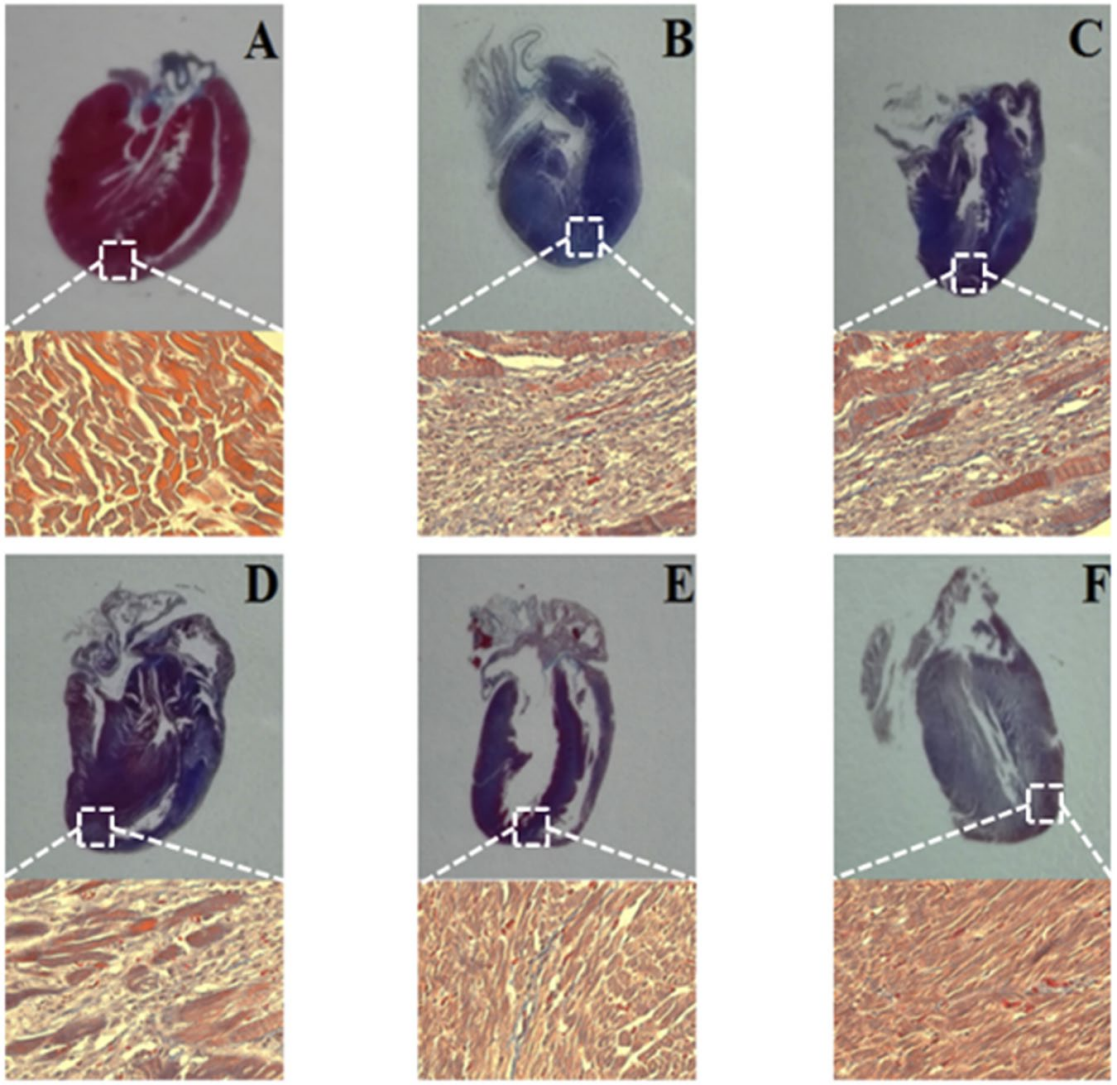
Histological sections of myocardium stained with Masson’Trichrome, and their quantitative analysis. Fibrosis evaluated based on a histological analysis with Masson’s trichrome staining, blue staining indicates fibrosis (**A**–**F**, represent results of Sham, MIRI, L-TFDM, M-TFDM, H-TFDM, Control drug group, respectively, n = 10).

Masson’s trichrome staining performed 30 days after the pretreatment showed that H-TFDM and trapidil tablets groups markedly reduced cardiac fibrosis induced by MIRI (Fig. 7), verifying the long-term protective effects of TFDM on infarcted myocardium. Tissue from the Sham group showed loss of myocardial structure without collagen fibers, indicating severe edema between the myocardial cells, with a woven myocardial structure observed. Masson’s trichrome staining of longitudinal sections of the heart revealed a large fibrotic area (blue) in the MIRI group. The fibrotic areas were significantly smaller in the H-TFDM and trapidil tablets groups.

## Discussion

Many drugs protect against MIRI. However, their use is hindered by side effects, drug safety and ethical concerns. An optimal pharmaceutical means of cardio-protection has yet to be found. A number of natural anti-inflammatory, anti-oxidative and anti-apoptotic substances have been recently described as safe, non-toxic and viable treatment options. Regarding MIRI, oral administration of particularly an anti-apoptotic natural substance would be therapeutically effective and might boost patient compliance^36–38^. We have previously reported that flavonoids from *D. moldavica* L. are efficient free radical scavengers with anti-oxidative and cardio-protective properties *in vitro*^30^. These compounds may prevent MIRI by blocking free radicals formed after reperfusion^39^.

As critical myocardial enzymes, LDH and CK-MB can be employed to assess the degree of myocardial injury. In our study, H-TFDM group significantly reduced levels of LDH and CK-MB. This indicates that H-TFDM may protect against MIRI. Additionally, H-TFDM resulted in the reduction of MDA as well as improved SOD activity, which can confirm the anti-oxidative characteristics of TFDM^35,38^.

Extensive clinical and animal studies have highlighted that apoptosis severely affects MIRI, resulting in a loss of cardiomyocytes^40,41^. The death receptor Bcl-2/Bax can be used to mediate the extrinsic apoptotic pathway, which includes the activation of caspase-7 and caspase-3^42,43^. The activation of caspase-9 and dysfunction of it are involved in the intrinsic pathway^38^. Ischemia, especially when it is mixed with reperfusion, leads to the translocation of Bax with the outer mitochondrial membrane. This demonstrates the connection with the reduced Bcl-2/Bax and the enhanced expression levels of Bax^44^. Bcl-2 inhibits apoptosis and decreases the infarct area in the heart following MIRI^45,46^. The caspase family also plays a pivotal role in apoptosis^47,48^. Presently, the expression of Bcl-2 (anti-apoptotic protein), Bax (proapoptotic protein), cleaved caspase-3 and caspase-7 and caspase-9 activity in MIRI rats were detected. TFDM pre-treatment made significant increase of Bcl-2 and reduced Bax, corresponding with a raised Bcl-2/Bax ratio, and increased levels of cleaved caspase-3, caspase-7 and caspase-9 protein.

The Bcl-2 and Bax proteins are essential in deciding cell survival or death after apoptosis is induced in the apoptotic pathway^37^. How TFDM regulates the expression of the apoptotic proteins Bcl-2 and Bax in oxidative stress is unclear. We suggest that the antioxidant action of TFDM may be responsible, since anti-oxidants influence apoptosis-related genes in cells during oxidative stress. Alternatively, the activation of PI3K and ERK1/2 may be related to gene expression in the Bcl-2 family^49,50^. Indeed, the activation of PI3K and Akt may reduce cell apoptosis and promote survival^51^.

Studies have confirmed that cardiac protective mechanisms involve signaling through phosphorylation of reperfusion injury salvage kinase (RISK) pathway components, including Akt and ERK1/2, and the inactivation of GSK-3*β*^52,53^. Several downstream proteins of Akt may be regulatory molecules of cell survival. These include GSK-3*β*, Bcl-2 and Bax^54^. However, the molecular mechanism for the effects of TFDM on RISK pathway is still unknown. The present data provide insight into the mechanism potential cardio-protection of TFDM for MIRI. TFDM activated signal transduction by both the PI3K/Akt/GSK-3*β* and ERK1/2 pathways. Elsewhere, it was reported that there has a blockage of the ERK1/2 signaling pathway effectively inhibits the PI3K/Akt/GSK-3*β* signaling pathway^55^. However, we did not observe any influence of ERK1/2 inhibitor on the phosphorylation of the GSK-3*β* in MIRI rats, which might contribute to a synergistic effect of the five flavonoid components of TFDM. Meanwhile, pre-treatment of H-TFDM obviously increased Akt and GSK-3*β* phosphorylation expression *in vivo*. Akt has cardioprotective effects involving the phosphorylation of diverse target molecules, such as GSK-3*β*, which preserves mitochondria integrity^56^. Indeed, Akt protective survival signaling mediates different functions of mitochondria. In addition, the cardioprotective effects of Akt could involve blocked opening of the permeability transition pore of mitochondria to preserve mitochondrial integrity^11,57,58^. In the MIRI rat models, overexpression and strengthened activity of Akt are related to increased phosphorylation of GSK-3*β*^59,60^. Inhibition of GSK-3*β* by Ser9 phosphorylation suppresses mitochondrial permeability transition pore (mPTP) opening and enhances cell survival by inducing local infarct size limitation against MIRI. At the same time, ERK1/2 is a protective factor against myocardial infarction and MIRI^61^. ERK1/2 activation plays a pivotal role in the process of oxidative stress and apoptosis, promotes cell survival and protects from impaired cardiac function and cardiac injury in MIRI heart^62^. The phosphorylation of ERK1/2 is beneficial for myocardial apoptosis and recovery of cardiac function. Activation of the PI3K/Akt and the ERK1/2 pathways inhibits the conformational change of Bax that is required for its translocation to the mitochondria and subsequent, inhibition of apoptosis. In addition, the up-regulation of ERK1/2 and PI3K/Akt inhibits the caspase cascade, such as caspase-3 caspase-7 and caspase-9, etc.

Phosphorylation of Akt inhibits apoptosis and promotes cell viability in MIRI rats^63^. Presently, to determine whether activation of the PI3K/Akt pathway made mechanical to TFDM-induced cardio-protection, we used LY294002 (a specific target inhibitor of PI3K), prior to reperfusion. LY294002 obviously inhibited anti-apoptotic activity of TFDM, indicating that the anti-apoptotic activity of TFDM is PI3K/Akt-dependent. TFDM enhanced the expression level of Akt of phosphorylation in contrast to the MIRI group. Moreover, TFDM pretreatment was abolished by using PD98059 (an ERK1/2 inhibitor). TFDM could increase ERK1/2 phosphorylation, whereas, PD98059 could inhibit the activation of ERK1/2. These findings suggest that activation of the ERK1/2 pathway is needed for the myocardial protective effects of TFDM pretreatment. Thus, TFDM pretreatment may act mainly to reduce myocardial apoptosis induced by MIRI via the PI3K/Akt/GSK-3*β* and ERK1/2 signal pathways.

Accumulating evidence suggests that there are a variety of bioactive components contribute to TFDM’s cardioprotection. These include tilianin, acacetin-7-O-β-D-glucuronide, diosmetin-7-O-β-D-glucuronide, apigenin-7-*O*-β-D-glucuronide and luteolin-7-*O*-β-D-glucuronide. They are all cardioprotective^30^. Their water solubility makes them easy to dissolve and administer, and increases their bioavailability *in vivo*. Yet, these compounds have not been studied well and have not been therapeutically developed compared to other compounds. In addition, the greater number of hydroxyl substituents on the backbone structure, the stronger the antioxidant activity of the flavonoid glycoside. The anti-oxidative activity in the membrane system depends on the number of hydroxyl groups and the polarity and hydrophobicity of the tested compounds. The O-H bond dissociation enthalpy, C ring’s spin density and stable molecular structure, presence of flavonoid glycoside, ortho-hydroxy in the B-ring, 2,3-double bond and 4-carbonyl moiety contribute to a more balanced distribution of spin density, reducing the bond dissociation enthalpy of O-H in the B-ring and increasing free radical scavenging activity of compounds. The five flavonoid glycosides in TFDM consistent with above molecular characteristics include two benzene rings on either side of a heterocycle containing three additional carbon atoms and are very similar to the structure of strong antioxidants including rutin, linarin and scutellarin.

**Figure 8:**
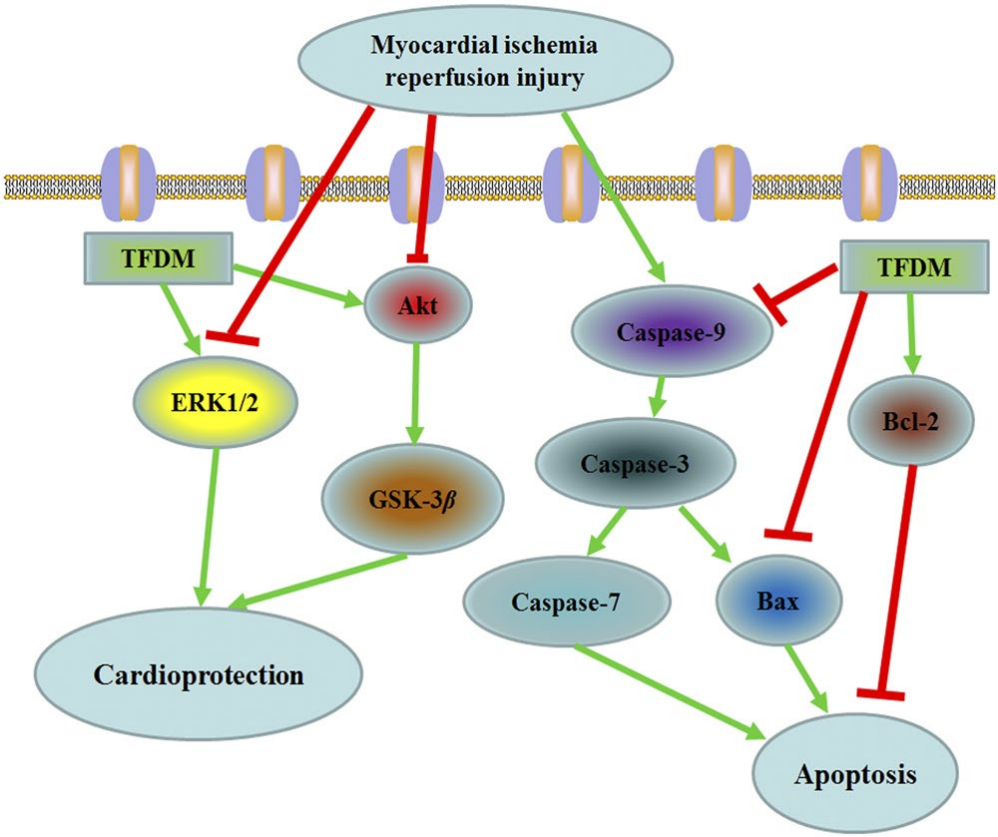
Putative mechanism of TFDM improved myocardial apoptosis.

Generally, the existing studies focus on the possible mechanism of TFDM against MIRI and the *In vivo* cardio-protective function. It is shown from the data that the preservation of the myocardial injury can be prevented through TFDM against the apoptosis induced by MIRI through the regulation of the proteins related with apoptosis, which include caspase-9, caspase-7, caspase-3, Bax and Bcl-2 through the activation of PI3K/Akt/ERK1/2 and GSK-3*β*, signaling pathways and also through the enhancement of the anti-oxidant enzyme mechanisms. Therefore, cardio-protection can be provided by TFDM through the anti-apoptotic signaling pathways (Fig. 8). This actually is a kind of RISK pathway, in which the MIRI is attenuated. Given the long-term treatment, it can resolve the safety issues successfully. The progression of chronic cardiovascular conditions can be prevented or delayed by TFDM because of its oral bioavailability.

## Materials and Methods

### Materials

The medicinal plant *D. moldavica* L. was collected in FuKang (Xinjiang, China) by Prof. Jian-guo Xing is from the Xinjiang Institute of Materia Medica. Prof. Cheng-hui He established the authenticity of the material, who came from the Xinjiang Institute of Materia Medica (Voucher number: D140811). Radio-immunoprecipitation assay (RIPA) lysis buffer was purchased from Beyotime Institute of Biotechnology (Beijing, China). Terminal Deoxynucleotidyl Transferase-Mediated dUTP Nick End-Labeling (TUNEL) was obtained from Boehringer (Mannheim, Germany). TTC were purchased from Sigma-Aldrich (St. Louis, MO, USA). Commercial cell death detection kit was obtained from Boster Co., Ltd., (Beijing, China). LY294002 (PI3K inhibitor) was purchased from Abcam (Cambridge, MA, USA). PD98059 (ERK1/2 inhibitor) was purchased from Sigma-Aldrich (St. Louis, MO, USA).

### Experimental Animals

In this study, male 10–12 weeks old SD rats at the start of the experiment were used (provided by the Department of Experimental Animal Center in Xuanwu Hospital of Capital Medical University). All animals were maintained under standard condition (22 ± 2°C temperature and 12-h light-dark cycle) with water and rodent chow available *ad libitum*. The Department of Experimental Animal Center in Xuanwu Hospital of Capital Medical University supported this study. Additionally, all animal experiments were in strict accordance with the National Institutes of Health Guide for the Care and Use of Laboratory Animals.

### TFDM Preparation

The preparation of TFDM was finished by the Xinjiang Institute of Materia Medica. Under the room temperature, the extraction of *D. moldavica* L. (50 g) was done three times in 40% ethanol (500 mL). Afterwards, it is followed by a heating-refluxing extraction lasting for 2 h. The extracted solution was filtered through a 180-mesh sieve. The crude extract was sequentially extracted with 500 mL petroleum ether, dichloromethane, ethyl acetate, methanol, *n*-butanol and water. The extracted solvent was evaporated (40 °C) and dried. The chemical constituents of TFDM were determined by high performance liquid chromatography (HPLC) analysis as summarized in Fig. 1 and Table 2.

**Table 2:** HPLC analysis of TFDM.

Peak	Compound name	Formula	Ratio in subfraction
1	Luteolin-7-*O*-β-D-glucuronide	C_21_H_17_O_12_	21.62
2	Apigenin-7-*O*-β-D-glucuronide	C_21_H_18_O_11_	7.53
3	Diosmetin-7-O-β-D-glucuronide	C_22_H_20_O_12_	34.43
4	Acacetin-7-O-β-D-glucuronide	C_22_H_20_O_10_	6.22
5	Tilianin	C_22_H_22_O_10_	28.15

### Animal Models and Experimental Groups

Healthy SD male rats (280–320 g) were randomly divided into eight groups. All rats were fed with drug or normal saline by gavage (10 mL/kg, once day) for two weeks before surgery. After the last administration, surgery was done 15 min. There were eight experimental groups. The Sham group received normal saline (10 mL/kg/day, orally, n = 40), with no ischemia or reperfusion. The Model group received normal saline (10 mL/kg/day, orally, n = 40). The positive control drug group: rats was pretreated with trapidil tablets (13.5 mg/kg/day, n = 30); TFDM solution groups were pretreated with L-TFDM (3 mg/kg/day), M-TFDM (10 mg/kg/day) and H-TFDM (30 mg/kg/day) (n = 30, 30 and 40).

**Figure 9:**
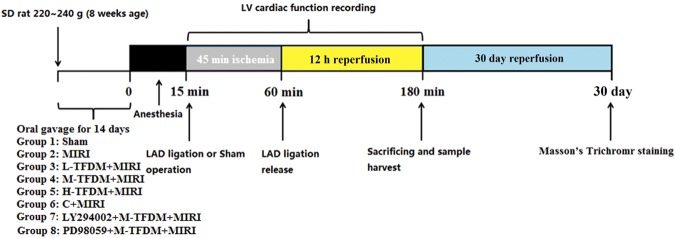
Experimental protocol to determine the effects of TFDM on MIRI in SD rats.

Meanwhile, we tested whether TFDM-induced expression of GSK-3*β* in the myocardium was inhibited by LY294002 (a specific inhibitor of PI3K) and expression of ERK1/2 in the myocardium was inhibited by PD98059 (a specific inhibitor of ERK1/2). The experimental animals were separated into five groups: rats in the Sham group, MIRI group, TFDM (30 mg/kg/d) + MIRI + LY294002 group (n = 10), TFDM (30 mg/kg/d) + MIRI + PD98059 group (n = 10) and TFDM (30 mg/kg/d) + MIRI group underwent the same operation as previously described. The LY294002 group received a single intravenous injection of LY294002 (7.5 mg/kg in 5% dimethylsulfoxide) 20 min before reperfusion. In the PD98059 group, the ERK1/2 inhibitor was administered in the same way as the LY294002 group (1 mg/kg). (Fig. 9).

Rats were anesthetized by an intraperitoneal injection of sodium pentobarbital (40 mg/kg,). All rats then underwent tracheotomy and tracheal intubation. Electrocardiograms (ECGs) were acquired continuously using a MP150 apparatus (BIOPAC Systems, Inc., Goleta, CA, USA) after placement of subcutaneous stainless steel electrodes connected to the electrocardiograph.

A left lateral thoracotomy was performed at the fourth intercostals space with an incision size that was sufficient to expose the pericardium. A 6-0 suture line was placed at the origin of the left coronary artery. Occlusion of coronary artery resulted in a visible blanched area in the myocardium distal to the ligation site, serving as an indicator for successful coronary artery ligation. Ischemia was further confirmed by electrocardiographic changes evident as ST segment elevation. After ischemia, the slipknot was unfastened, and the myocardium was reperfused for 12 h. After reperfusion for 12 h, rats were sacrificed and blood and heart tissue were collected.

### Myocardial infarction assessment and Hematoxylin & Eosin (H&E) Staining

After 12-h reperfusion, the 2 mL of 2% solution of Evans Blue dye was injected into the right jugular vein to identify the area prone to ischemic damage. Rat hearts were removed rapidly, washed, frozen, and cut into 5 pieces with cross-section. Heart tissues were incubated in 1% TTC at 37 °C for 15 min respectively. Then tissues were fixed in 10% formalin solution. The area of infarct size (IS) and area at risk (AAR) were quantitated using Image-Pro Plus 6.0 software (Media Cybernetics, Inc., USA). Then, the ratio of area of infarct size to area at risk was calculated. For H&E staining, heart tissues were fixed in buffered paraformaldehyde and embedded in paraffin. Histological changes of the heart tissues in different groups were investigated using H&E staining of 2 μm thick tissue sections by optical microscopy at 400× magnification. A previously described scoring system^64^ was used to assess myocardial injury in an uninformed subgroup of animals [0, no change, 1, minimum damage (focal myocyte damage); 2, mild damage (small multifocal degeneration with slight degree of inflammatory process); 3, moderate damage (extensive myofibrillar degeneration and/or diffuse inflammatory process) and 4, severe damage (necrosis with diffuse inflammatory process)]. The score from light microscopy observation was statistically analyzed using a nonparametric test.

### TUNEL staining of myocardial apoptosis

Myocardial tissues perpendicular to the long axis of heart midline were sliced with the thickness of 1–2 mm after reperfusion for 12 h. Samples were incubated with terminal deoxynucleotidyl transferase and detection buffer that was conjugated with horse-radish peroxidase. Two micrometer thick sections of cardiac tissue were used. The number of TUNEL-positive nuclei was counted and expressed as the percentage of the total number of cellular nuclei at 400× magnification. The formula used was:

$${\rm{A}}{\rm{p}}{\rm{o}}{\rm{p}}{\rm{t}}{\rm{o}}{\rm{t}}{\rm{i}}{\rm{c}}\,{\rm{i}}{\rm{n}}{\rm{d}}{\rm{e}}{\rm{x}}\,({\rm{ \% }})={\rm{N}}{\rm{u}}{\rm{m}}{\rm{b}}{\rm{e}}{\rm{r}}\,{\rm{o}}{\rm{f}}\,{\rm{T}}{\rm{U}}{\rm{N}}{\rm{E}}{\rm{L}}\,{\rm{p}}{\rm{o}}{\rm{s}}{\rm{i}}{\rm{t}}{\rm{i}}{\rm{v}}{\rm{e}}\,{\rm{c}}{\rm{e}}{\rm{l}}{\rm{l}}{\rm{s}}/{\rm{T}}{\rm{o}}{\rm{t}}{\rm{a}}{\rm{l}}\,{\rm{c}}{\rm{e}}{\rm{l}}{\rm{l}}\,{\rm{n}}{\rm{u}}{\rm{c}}{\rm{l}}{\rm{e}}{\rm{i}}\times 100$$

### Biochemical Studies

Blood was collected from the abdominal aorta and centrifuged at 3000 g and 4 °C for 10 min to isolate serum. Then, serum was determined and analyzed for contents of LDH and MDA and SOD activity by spectrophotometrically via commercial assay kits and serum CK-MB was quantified using a commercially available enzyme linked immunosorbent assay (ELISA) kit, according to the manufacturer’s protocol.

### Ultrastructural Analysis

The affected region was used for the collection of the heart tissues, which were fixed via the 2.5% glutaraldehyde. Later, it was washed through the phosphate buffered solution (PBS, 0.1 M, pH 7.4). Afterwards, it was fixed under 4 °C lasting for 2 h via the 1% osmium tetroxide. The araldite was used to embed the samples while then it was sliced to ultrathin sections (70–80 nm). The lead acetate and 1% uranyl acetate were used to stain the sections. Later, it was examined through the TEM, namely the transmission electron microscopy via the H-600 microscope (Hitachi, Tokyo, Japan) when the magnifications were between ×15,000 upward to evaluate the ultrastructural characteristics of cardiomyocytes.

### Western Blot Analysis

50 μg protein was extracted from the homgenation of the tissue, which was put and boiled in the loading buffer, in which 1% of phenylmethanesulfonyl fluoride (PMSF) was used, the process of which lasted for 10 min. Then, it was separated by sodium dodecyl sulfate-polyacrylamide gel electrophoresis (SDS-PAGE) on correspondingly 8% and 12% gels. Later, there was a transfer of the resolved proteins to the PVDF, namely the polyvinylidene difluride membrane (Millipore, Bedford, MA, USA). The TBST, namely Tris buffered saline containing Tween was used for the washing of the membranes, which lasted for 10 min. The observation was conducted through the protein bands. Afterwards, the protein Marker and Pierrexon stain were used for the cropping of the respective protein bands. Finally, the membranes were blocked with 5% non-fat dry milk, and incubated overnight at 4 °C with corresponding primary antibody. Then, the membranes were incubated with secondary antibodies at a 1:2000 dilution for 2 h at room temperature. The protein bands were detected using an electrochemiluminesence (ECL) system and quantified by AlphaView SA 3.4.0.0 Software (ProteinSimple, San Jose, CA, USA). β-actin and GAPDH were used as internal standard.

### Long-term Effect of TFDM on Myocardial Fibrosis

After 30 days of the TFDM and control drug pretreatment, to figure out the fibrosis, the cardiac tissue of the rats of every group was fixed through the formaldehyde, which lasted between 6 to 12 h. After the paraffin embedding, 30 μm intervals were used to obtain the 4 μm-thick sections. The collagenous fibrosis of the trichrome of Masson was prepared by staining the sections.

### Statistical Analysis

The statistical software SPSS 16.0 (SPSS Inc., Chicago, IL, USA) was adopted to conduct the statistical analysis. All values were expressed as mean ± standard error. One-way analysis of variance (ANOVA), followed by Turkey’s test for multiple comparisons was performed to assess differences among groups. Besides, differences were considered significant when probability (*P*) < 0.01.

## Data Availability

All data generated or analyzed during this study are included in this article.
